# Reimbursement Status and Recommendations Related to Orphan Drugs in European Countries

**DOI:** 10.3389/fphar.2019.01279

**Published:** 2019-11-27

**Authors:** Ewa Stawowczyk, Krzysztof Piotr Malinowski, Paweł Kawalec, Rafał Bobiński, Jacek Siwiec, Dimitra Panteli, Helene Eckhardt, Steven Simoens, Antònia Agusti, Marc Dooms, Andrzej Pilc

**Affiliations:** ^1^Faculty of Health Sciences, University of Bielsko-Biala, Bielsko-Biała, Poland; ^2^Institute of Public Health, Faculty of Health Sciences, Jagiellonian University Medical College, Krakow, Poland; ^3^Department of Health Care Management, Berlin University of Technology, Berlin, Germany; ^4^WHO Collaborating Centre for Health Systems Research and Management, Berlin, Germany; ^5^Research Hub of the European Observatory on Health Systems and Policies, Berlin, Germany; ^6^Department of Pharmaceutical and Pharmacological Sciences, KU Leuven, Leuven, Belgium; ^7^Clinical Pharmacology Service, Catalan Institute of Pharmacology Foundation, Vall d’hebron University Hospital, Barcelona, Spain; ^8^Department of Pharmacology, Therapeutics and Toxicology, Universitat Autònoma de Barcelona, Barcelona, Spain; ^9^University Hospitals Leuven, Leuven, Belgium; ^10^Institute of Pharmacology, Polish Academy of Sciences, Krakow, Poland

**Keywords:** health technology assessment, drug policy, rare disease, reimbursement, orphan

## Abstract

**Objective:** To review the reimbursement recommendations issued by selected European health technology assessment agencies for orphan drugs and the reimbursement status of these drugs; to assess the relationship between the type of recommendation and reimbursement status.

**Methods:** The list of orphan drugs to be included in the analysis was obtained from the European Medicines Agency and Orphanet. Seven European states were included in the analysis: Belgium, England, France, Germany, Poland, Scotland, and Spain. For all identified orphan drugs, relevant data on the reimbursement status and type of recommendation were collected for each country. The relationship between the type of recommendation and reimbursement status was evaluated separately for each considered country, using Cohen’s kappa coefficient for the measurement of agreement; sub-analyses for oncology and metabolic drugs were performed.

**Results:** Most reimbursement recommendations for orphan drugs were positive (71%), while approximately 17% were negative and almost 13% were conditional. The highest percentage of positive reimbursement recommendations was observed in Spain (97%) and France (95%) and the highest percentage of negative reimbursement recommendations was revealed for Poland (49%). On average, 65% of the 163 analyzed orphan drugs were reimbursed from public funds. The highest number of reimbursed orphan drugs was observed in Germany (*n* = 148), while the lowest, in Poland (*n* = 41). Considering all analyzed drugs, the highest agreement between recommendations and reimbursement status was observed for Spain (value of 1), and the lowest, for Germany (κ = -0.03).

**Conclusions:** On average, more than 60% of identified orphan drugs were reimbursed from public funds in the included countries, and the majority of reimbursement recommendations were found to be positive. The agreement between reimbursement recommendations and reimbursement status differed between the countries, but overall, it did not show any patterns, as it ranged from -0.03 to 1 (κ coefficient).

## Background

From the point of view of epidemiology, diseases may be divided into common, rare, and ultra-rare. First and foremost, there is no uniform criterion for defining rare diseases, applicable across countries.

The criterion used in the European Union (EU) assumes that a rare disease affects not more than 5 out of every 10,000 people, which corresponds to a population of approximately 253,000 EU residents (https://www.ema.europa.eu/en/news/development-medicines-rare-diseases). In line with the definition of the World Health Organization, a rare disease is a disease which affects, as a maximum, 65 out of 100,000 people, whereas the Swedish National Board of Health and Welfare defines a rare disease as a disease which affects not more than 10 out of 100,000 people in a population. In the United States (US), the Orphan Drug Act (1983) features a provision that a rare disease affects fewer than 200,000 residents in the US (i.e., not more than approx. 7 cases per 10,000 patients) (Łanda et al., 2009). In Australia and Japan, a rare disease is defined as affecting 11 and 40 people out of 100,000 people, respectively (Denis et al., 2009). Similarly, there are no commonly adopted international or European definitions for ultra-rare diseases. In England, this concept entails a disease with a prevalence of 1 case per 50,000 people (Łanda et al., 2009), while in Poland, an ultra-rare disease is a disease which affects not more than 750 people in the population (Zarządzenie Nr 17/2007 Prezesa Narodowego Funduszu Zdrowia z dnia 10 kwietnia 2007 r. w sprawie zasad wdrażania terapeutycznych programów zdrowotnych finansowanych przez Narodowy Fundusz Zdrowia, 2007). The value mentioned above corresponds to the definition of an ultra-rare disease adopted in England, i.e., 1:50,000 people.

An orphan drug is a medicinal product that is developed to treat, diagnose, or prevent a specific rare disease (European Medicines Agency, 2018). In recent decades, more and more medicines have been approved for rare indications. Additionally, special programmes have been developed to better diagnose, prevent, and treat those conditions (World Health Organization).

However, many rare diseases stay without treatment. Medicines for which the targeted population is small are commercially unattractive for the pharmaceutical industry. Many orphan drugs would not be developed and authorized without additional incentives (Franco, 2013). The EU regulatory framework allows marketing authorization holders for orphan drugs to: 1) obtain scientific advice on clinical trial protocols at a reduced charge, 2) gain access to the European Medicines Agency centralized licensing procedure, 3) get reduction of registration costs, and 4) benefit from 10 years of market exclusivity after registration (European Medicines Agency, 2018).

Clinical development and market authorization of orphan drugs is not enough. Orphan drugs must also be reimbursed from public funds to be accessible to patients, particularly as their prices are usually significantly higher than those of drugs for common diseases. Reimbursement decisions are increasingly based on health technology assessment (HTA), usually entailing economic evaluation. HTA agencies issued reimbursement recommendations which may be positive, negative, or conditional (positive after fulfilling additional conditions). Based on the HTA agency recommendations, decision makers, responsible for drug reimbursement, produce a final decision on reimbursement which may be positive or negative. Positive reimbursement recommendation does not guarantee reimbursement, and similarly negative reimbursement recommendation does not always mean a lack of reimbursement. Economic evaluations of orphan drugs are particularly difficult as available clinical data are limited and there are usually no relevant comparators. Resource use and costs of treatment are also challenging to calculate because very few clinical centers are specialized in diagnosing and treating rare diseases—collected values may be non-representative, and for many countries even not available. Due to their high prices, orphan drugs usually have a very high incremental cost-effectiveness ratio (ICER), which typically surpasses willingness-to-pay thresholds set in different systems and may lead to negative reimbursement recommendations issued by HTA agencies and an exclusion from reimbursement. Some countries set different, higher thresholds for orphan drugs than for medicines used in common conditions. The example of such country is the Netherlands, where no drugs have been excluded from coverage because of their unfavorable cost-effectiveness (Carrera and IJzerman, 2016). Governments may decide to reimburse orphan drugs despite a negative recommendation, if they constitute the only therapeutic option for a selected group of patients (Kawalec et al., 2018).

To provide an overview of the availability of orphan drug funding in statutory health systems in Europe, this study aimed to review reimbursement recommendations issued by European HTA agencies for orphan drugs and the reimbursement status of these drugs. This study will also answer the question if reimbursement recommendation of considered HTA agency for orphan drug corresponds with reimbursement status. Additionally, the subgroups of drugs registered in oncological and metabolic indications were analyzed separately.

## Methods

The full list of orphan drugs to be included in the analysis was obtained from the website of the European Medicines Agency (2018) and Orphanet (2018). All orphan drugs registered till end of July 2018 by the European Medicines Agency (EMA) were included. The following European states, for which both reimbursement and recommendation data were available, were included in the analysis: England, France, Germany, Poland, Scotland, and Spain. Additionally, Belgium was included, but only the reimbursement status was available for this country. The countries were selected to represent a mixture of health system financing modalities and expected availability of reimbursed orphan drugs. Information on reimbursement status and type of recommendation was collected by experts in each included country using publicly available information (**Supplementary Table 1**). Schematic figure on methodology was presented in **Supplementary Figure 1**.

Drugs with multiple rare indications were treated as one observation (all indications of the drug treated as one). Reimbursement recommendations were coded as follows: negative—reimbursement from the public fund is not recommended, positive—reimbursement from public fund is recommended, or conditional—reimbursement is recommended but only if additional conditions are met. This coding was not directly applicable for Germany, as new medicines are generally automatically reimbursable upon marketing authorization; this is picked up further in the Discussion section. Only the reimbursement status was assessed, but no information on drug availability in each country was collected. The data on the type of reimbursement recommendations and reimbursement status were summarized with counts and percentages. As more than 30% of the orphan drugs included in the analysis were registered in oncological indications, and about 20% were registered in metabolic indications, those subgroups of drugs were also analyzed separately.

As reimbursement policy and HTA agency guidelines for orphan drugs differ among European countries, the relationship between the type of recommendation and reimbursement status was assessed separately for each country. The agreement between recommendations and reimbursement status for each country separately as well as between countries (for both recommendations and reimbursement status) was assessed using Cohen’s kappa coefficient of agreement (*κ*) for the measurement of agreement. The *κ* coefficient can range from -1 to 1, with values lower than 0 denoting no agreement, 0 representing the amount of agreement by random chance, and 1 denoting perfect agreement. The values between 0.01 and 0.20 denote slight agreement, between 0.21 and 0.40—fair agreement, between 0.41 and 0.60—moderate agreement, between 0.61 and 0.80—substantial agreement, and between 0.81 and 0.99—almost perfect agreement (Cohen, 1960). All *κ* coefficients were supported with 95% confidence intervals and rounded to two decimal places.

## Results

Overall 163 orphan drugs were identified in EMA and Orphanet databases, including 54 drugs with oncological indication and 33 drugs with metabolic indication; the list was valid for July 2018 (**Supplementary Table 2**).

### Reimbursement Recommendations for Orphan Drugs

**Figure 1:**
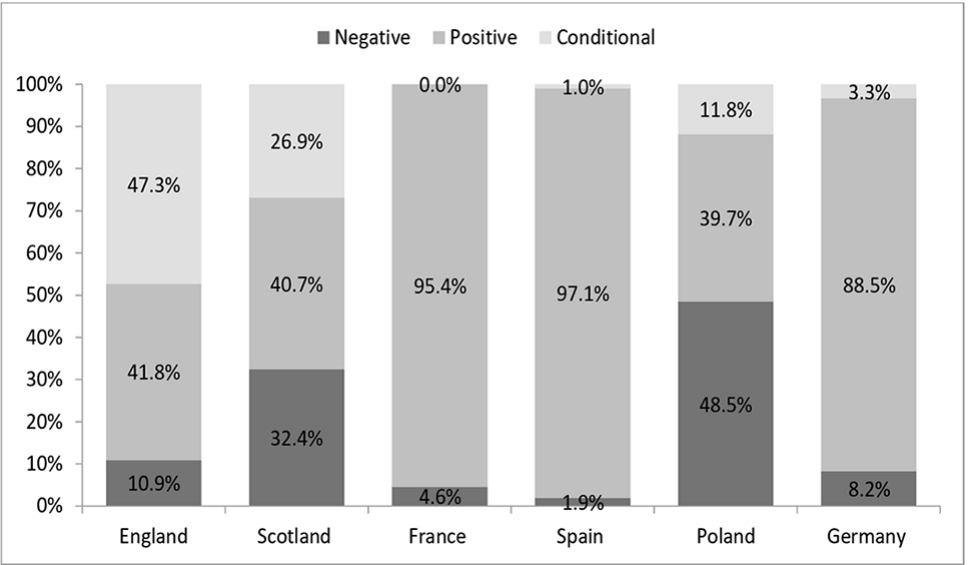
Percentage of positive, negative, and conditional reimbursement recommendations for orphan drugs (all) in analyzed countries

We identified 526 reimbursement recommendations, which were issued for the analyzed orphan drugs in all countries. The highest number of recommendations was identified in France (*n* = 131), Scotland (*n* = 108), and Spain (*n* = 103). Considering all states, the majority of reimbursement recommendations for orphan drugs were positive (71%), while 17% were negative and almost 13% were conditional. The highest percentage of positive reimbursement recommendations was observed in Spain (97%) and France (95%). In England there was a similar percentage of positive and conditional reimbursement recommendations—42% and 47%, respectively. The highest percentage of negative reimbursement recommendations was observed in Poland (49%) and Scotland (32%). In France, the HTA agencies issued no conditional reimbursement recommendations for orphan drugs (**Figure 1**).

About 41% (216 out of 526) of reimbursement recommendations were issued for orphan drugs used for oncologic indications. The highest percentage of recommendations for oncologic drugs among all recommendations for orphan drugs was observed in England (63.6%), Poland (44.1%), and Scotland (40.7%), while the lowest, in France (35.1%). Considering all analyzed countries, the majority of reimbursement recommendations for oncologic orphan drugs were positive (69%), while 16% were negative and 15% were conditional. The highest percentage of positive reimbursement recommendations for oncologic orphan drugs was observed in France (96%) and Spain (95%), while the lowest, in Poland (40%) (**Table 1**).

**Table 1:** Recommendation for oncologic and metabolic drugs.

Drugs	Recommendation	England	Scotland	France	Spain	Poland	Germany	All
Oncologic	Negative	3 (8.6%)	11 (25.0%)	2 (4.3%)	1 (2.6%)	15 (50.0%)	2 (8.7%)	34 (15.7%)
	Positive	17 (48.6%)	22 (50.0%)	44 (95.7%)	36 (94.7%)	12 (40.0%)	19 (82.6%)	150 (69.4%)
	Conditional	15 (42.9%)	11 (25.0%)	0 (0%)	1 (2.6%)	3 (10.0%)	2 (8.7%)	32 (14.8%)
Metabolic	Negative	2 (25.0%)	9 (56.3%)	1 (4.0%)	0 (0%)	6 (66.7%)	0 (0%)	18 (20.2%)
	Positive	4 (50.0%)	5 (31.3%)	24 (96.0%)	18 (100%)	3 (33.3%)	13 (100%)	67 (75.3%)
	Conditional	2 (25.0%)	2 (12.5%)	0 (0%)	0 (0%)	0 (0%)	0 (0%)	4 (4.5%)

About 17% (89 out of 526) of reimbursement recommendations were issued for orphan drugs used for metabolic indications. The percentage of recommendations for metabolic drugs among all recommendations for orphan drugs was similar between the countries. Considering all analyzed countries, the majority of reimbursement recommendations for metabolic orphan drugs were positive (75%), while 20% were negative and 4% were conditional. The highest percentage of positive reimbursement recommendations for metabolic orphan drugs was observed in Spain, Germany (100%; see caveat on the German system in the Discussion section, below), and France (96%), while the lowest, in Scotland (31%) and Poland (33%) (**Table 1**).

### Reimbursement Status for Orphan Drugs

**Figure 2:**
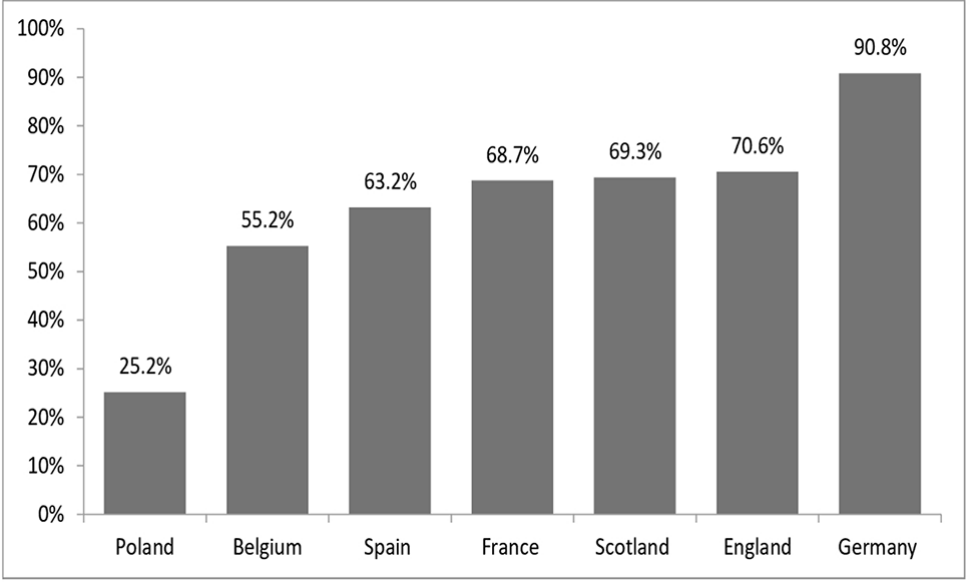
Percentage of reimbursed orphan drugs (all) in analyzed countries.

The reimbursement status was assessed for 163 orphan drugs. On average, 65% of analyzed orphan drugs were reimbursed from public funds. The highest number of reimbursed orphan drugs was observed in Germany (*n* = 148, 90.8%), England (*n* = 115, 70.6%), Scotland (*n* = 113, 69.3%), and France (*n* = 112, 68.7%), while the lowest, in Poland (*n* = 41, 25.2%) (**Figure 2**). Out of the 163 orphan drugs, 54 (33%) were used for oncologic indications. On average, 69% of analyzed oncologic orphan drugs were reimbursed from public funds, with the highest percentage observed in Germany (89%), France (80%), England and Scotland (72%), while the lowest, in Poland (31%). More than 20% of the analyzed orphan drugs were used for metabolic indications. On average, 64% of analyzed metabolic orphan drugs were reimbursed from public funds, with the highest percentage observed in Germany (97%), England and Scotland (82%), while the lowest in Poland (21%) (**Table 2**).

**Table 2:** Reimbursement status for oncologic and metabolic drugs.

Drugs	Reimbursement status	England	Scotland	France	Spain	Poland	Germany	All
Oncologic	Not Reimbursed	15 (27.8%)	15 (27.8%)	11 (20.4%)	17 (31.5%)	37 (68.5%)	6 (11.1%)	(31.2%)
	Reimbursed	39 (72.2%)	39 (72.2%)	43 (79.6%)	37 (68.5%)	17 (31.5%)	48 (88.9%)	(68.8%)
Metabolic	Not Reimbursed	6 (18.2%)	6 (18.2%)	12 (36.4%)	14 (42.4%)	26 (78.8%)	1 (3.0%)	(32.8%)
	Reimbursed	27 (81.8%)	27 (81.8%)	21 (63.6%)	19 (57.6%)	7 (21.2%)	32 (97.0%)	(67.2%)

### Agreement in Reimbursement Status Between Countries

Agreement in reimbursement status was assessed among all analyzed countries. The highest κ coefficient of agreement for all analyzed drugs was 0.91, observed between England and Scotland, and the lowest was 0.07, observed between Poland and Germany (**Table 3**; see caveat on the German system in the Discussion section). Considering only oncologic orphan drugs, the highest agreement (κ = 0.82) was observed between England and Scotland. The lowest agreement (κ = 0.11) was observed between England and Spain, Scotland and Poland, as well as Poland and Germany (**Table 4**). For metabolic drugs, the highest agreement (κ = 1) was observed between England and Scotland, and the lowest (-0.07), between Spain and both England and Scotland (**Table 5**).

**Table 3:** Agreement in reimbursement status between countries for all analyzed drugs.

	Scotland	France	Spain	Poland	Germany
**England**	0.91 (0.84–0.98)	0.38 (0.22–0.53)	0.17 (0.02–0.33)	0.18 (0.10–0.27)	0.17 (0.03–0.31)
**Scotland**		0.33 (0.17–0.48)	0.21 (0.05–0.36)	0.18 (0.09–0.27)	0.16 (0.02–0.30)
**France**			0.52 (0.39–0.66)	0.22 (0.13–0.31)	0.29 (0.15–0.44)
**Spain**				0.33 (0.23–0.42)	0.23 (0.11–0.36)
**Poland**					0.07 (0.03–0.10)

**Table 4:** Agreement in reimbursement status between countries for oncologic drugs.

	Scotland	France	Spain	Poland	Germany
**England**	0.82 (0.64–0.99)	0.40 (0.12–0.67)	0.11 (-0.16 to 0.39)	0.17 (0.00–0.35)	0.26 (-0.01 to 0.54)
**Scotland**		0.30 (0.01–0.58)	0.20 (-0.07 to 0.48)	0.11 (-0.07 to 0.29)	0.26 (-0.01 to 0.54)
**France**			0.43 (0.17–0.69)	0.21 (0.08–0.34)	0.52 (0.22–0.82)
**Spain**				0.35 (0.18–0.52)	0.32 (0.07–0.58)
**Poland**					0.11 (0.02–0.20)

**Table 5:** Agreement in reimbursement status between countries for metabolic drugs.

	Scotland	France	Spain	Poland	Germany
**England**	1.00 (1.00–1.00)	-0.03 (-0.33 to 0.28)	-0.07 (-0.35 to 0.21)	0.02 (-0.12 to 0.17)	-0.05 (-0.15 to 0.04)
**Scotland**		-0.03 (-0.33 to 0.28)	-0.07 (-0.35 to 0.21)	0.02 (-0.12 to 0.17)	-0.05 (-0.15 to 0.04)
**France**			0.62 (0.35–0.89)	0.27 (0.07–0.46)	0.10 (-0.09 to 0.30)
**Spain**				0.33 (0.11–0.56)	0.08 (-0.07 to 0.23)
**Poland**					0.02 (-0.02 to 0.05)

### Agreement in Recommendations Between Countries

Agreement in recommendations was also assessed. The highest κ coefficient of agreement for all analyzed drugs was 0.57, observed between England and Scotland, and the lowest was –0.15, observed between Scotland and Germany (**Table 6**). Considering only oncologic orphan drugs, the highest agreement (κ = 0.72) was observed between England and Scotland, and the lowest (κ = -0.13), between Scotland and Germany (**Table 7**). The highest agreement for metabolic drugs was between England and Scotland as well as Scotland and Poland (κ = 0.55). Small number of drugs precluded us from the calculation of the agreement between other combinations of countries (**Table 8**).

**Table 6:** Agreement in recommendations between countries for all analyzed drugs.

	Scotland	France	Spain	Poland	Germany
**England**	0.57 (0.24–0.89)	-0.06 (-0.11 to -0.00)	-0.04 (-0.10 to 0.02)	0.11 (-0.04 to 0.27)	-0.08 (-0.16 to -0.00)
**Scotland**		-0.06 (-0.12 to 0.00)	-0.02 (-0.07 to 0.02)	0.30 (0.09–0.50)	-0.15 (-0.26 to -0.04)
**France**			-0.03 (-0.05 to -0.00)	0.06 (-0.02 to 0.15)	-0.08 (-0.14 to -0.02)
**Spain**				0.08 (-0.03 to 0.19)	-0.08 (-0.16 to -0.00)
**Poland**					0.08 (-0.12 to 0.28)

**Table 7:** Agreement in recommendations between countries for oncologic drugs.

	Scotland	France	Spain	Poland	Germany
**England**	0.72 (0.35–1.00)	-0.07 (-0.13 to -0.00)	-0.06 (-0.14 to 0.03)	0.12 (-0.04 to 0.29)	-0.07 (-0.16 to 0.03)
**Scotland**		-0.09 (-0.20 to 0.02)	-0.05 (-0.15 to 0.04)	0.19 (-0.01 to 0.39)	-0.13 (-0.26 to 0.00)
**France**			-0.04 (-0.09 to 0.02)	0.07 (-0.06 to 0.19)	-0.07 (-0.16 to 0.03)
**Spain**				0.06 (-0.06 to 0.19)	-0.09 (-0.21 to 0.04)
**Poland**					-0.05 (-0.33 to 0.23)

**Table 8:** Agreement in recommendations between countries for metabolic drugs.

	Scotland	France	Spain	Poland	Germany
**England**	0.55 (-0.16 to 1.00)	NA	NA	NA	NA
**Scotland**		NA	NA	0.55 (-0.16 to 1.00)	NA
**France**			NA	NA	NA
**Spain**				NA	NA
**Poland**					NA

NA not available; impossible to assess due to small number of cases.

### Agreement Between Recommendations and Reimbursement Status Within Countries

**Figure 3:**
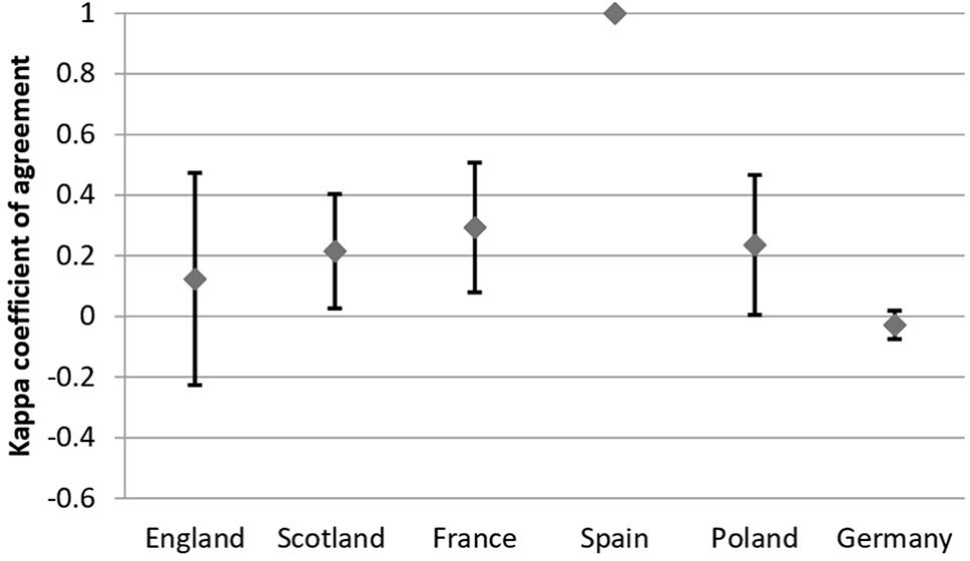
Agreement Between Recommendations and Reimbursement Status—all drugs.

**Figure 4:**
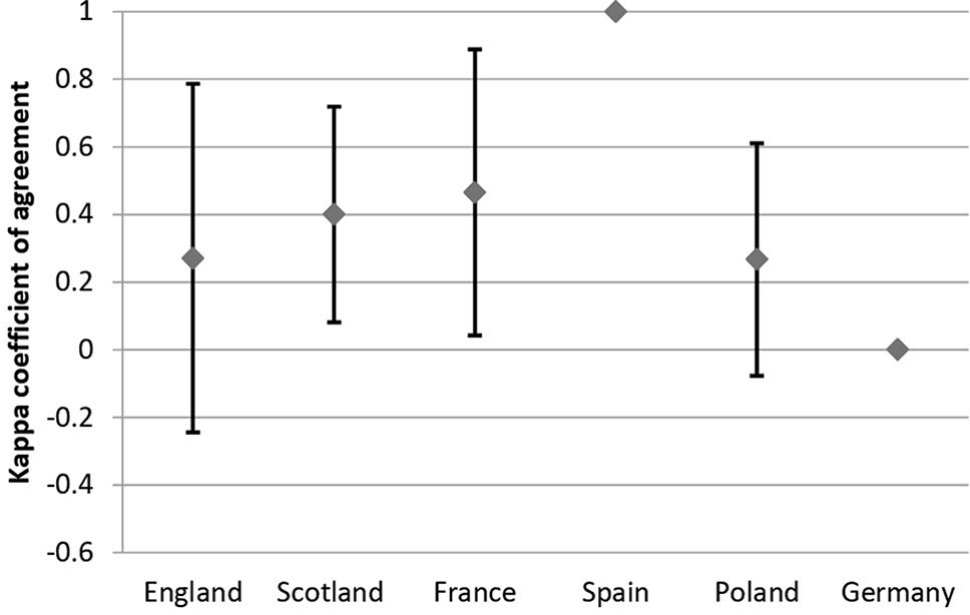
Agreement Between Recommendations and Reimbursement Status—oncologic drugs.

**Figure 5:**
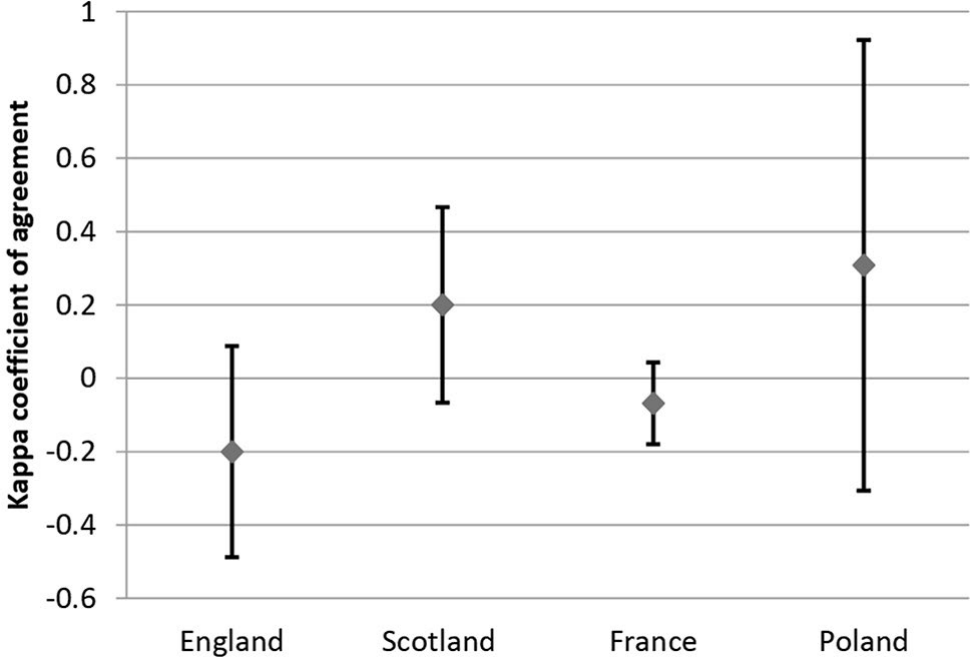
Agreement Between Recommendations and Reimbursement Status—metabolic drugs.

Considering all analyzed drugs, the highest κ coefficient of agreement between recommendations and reimbursement status was observed for Spain (κ = 1) and the lowest, for Germany (κ = -0.03; please refer to comment in Discussion) (**Figure 3**). For the subgroup of oncologic drugs, the corresponding κ values were 1 for Spain and 0 for Germany (**Figure 4**). For metabolic drugs, the corresponding κ values were 0.31 for Poland and -0.3 for England (**Figure 5**).

## Discussion

This study analyzed the type of reimbursement recommendations issued for orphan drugs in selected European countries, together with the reimbursement status. Most Western European countries were included in the analysis, but only one from Central Eastern Europe (CEE; Poland). The reason for that was because data on reimbursement status and reimbursement recommendations were not available in other CEE countries; Poland was the only CEE state with relevant data on drug reimbursement widely available online.

No detailed information on the reimbursement recommendations for individual orphan drugs was available in Belgium. Overall, in 2016, the Drug Reimbursement Committee issued 27% of positive and 27% of negative recommendations. The Committee was not able to issue any recommendations in the case of 9% of the assessed orphan drugs, and it advised to negotiate the managed entry agreement in 37% of cases. Taking those recommendations into account, the Minister issued positive decisions on the reimbursement of 73% of orphan drugs, and negative in 27%.

In Germany, new medicines are reimbursable upon entering in the market; manufacturers can set their price freely for the first year of circulation. Early benefit assessment of new active substances by the Federal Joint Committee (Gemeinsamer Bundesausschuss, G-BA) was introduced by the Pharmaceutical Market Restructuring Act (Arzneimittelmarktneuordnungsgesetz, AMNOG), which came into force on 1st January 2011. Drugs with new active substances undergo a benefit assessment based on an evidence dossier submitted by the manufacturer. The G-BA classifies the magnitude of the new drug’s added benefit compared to current best practice. This classification is the basis for the negotiation of the drug’s reimbursement price between the German Association of Statutory Health Insurance Funds and the manufacturer, which applies after the first year of circulation. In most cases, the reimbursement status remains unaffected. To allow for the special characteristics of orphan drugs, the AMNOG considers an added medical benefit of an orphan pharmaceutical as proven by means of the marketing authorization, unless its turnover at the expense of statutory health insurance exceeds €50 million in 12 months. If that mark is surpassed, a dossier has to be filed and a full benefit assessment takes place, followed by price negotiation.

In England, the assessment of orphan drugs for rare conditions is part of the Highly Specialised Technologies (HST) program. Following a consultation process in 2017, it was recommended that NHS England automatically fund drugs for ultra-rare conditions if the ICER of the drug is below £100,000 per QALY, a threshold which is substantially higher than the one usually applied for drugs (https://www.nice.org.uk/about/what-we-do/our-programmes/nice-guidance/nice-technology-appraisal-guidance/consultation-on-changes-to-technology-appraisals-and-highly-specialised-technologies). Technologies under the HST program are generally subject to a budget impact provision: if their budget impact exceeds £20 million in any of the first 3 years, negotiations between NHS England and the manufacturer may be initiated. The SMC in Scotland may consider at a Patient and Clinician Engagement (PACE) meeting further aspects of the new active substance for treatment of a rare disease, which were not part of the submitted manufacturer dossier (https://www.scottishmedicines.org.uk/how-we-decide).

Given the common framework of the NHS in England and Scotland, it is not surprising that recommendations and decisions on reimbursement status and between these countries achieve the highest agreement. Inversely, the particularity of the German reimbursement system, wherein (orphan) drugs are automatically reimbursed unless explicitly excluded, probably accounts for the lack of agreement regarding the recommendations decisions between Germany and the other countries. Generally, differences in both reimbursement recommendations and status may be due to different agency-specific evidentiary, risk and value preferences, or stakeholder input (Nicod, 2017).

Although some research projects were conducted, there is not a similar study evaluating the current situation in various EU countries. A study published by Zelei et al. (2016), who reviewed scientific evidence on the HTA for orphan drugs decision-making with a special focus on public payers in CEE countries, was identified. The authors revealed that CEE countries are more budget-restricted than Western European countries and could be more affected by the lack of clinical evidence for orphan drugs, which generally gain marketing authorization earlier than non-orphan drugs.

Szegedi et al. (2018) revealed that the highest expenditure on orphan drugs from 2013 to 2014 was observed in Belgium (€245–280 million), and the lowest, in Bulgaria (€8.3–12.2 million). The number of accessible orphan products, also observed in this study, suggests an equity gap between Eastern and Western Europe. The spending on orphan drugs as a proportion of the gross domestic product (GDP) as well as of public pharmaceutical and healthcare expenditures was lower in poorer countries, which indicates a substantial inequity in terms of patient access to orphan drugs, favoring higher-income countries.

Picavet et al. (2012) analyzed access to orphan drugs in almost all EU countries (except for Cyprus, Malta, and Portugal) based on data from IMS Health (2011). They showed that employing an HTA process has an important role in improving patients’ access to reimbursed orphan drugs, particularly in low-GDP countries. However, nowadays more low-GDP countries use a formal HTA process than in 2011; HTA process has been shown to play an important role in improving patient access to reimbursed orphan drugs, particularly in low-GDP countries.

Kamusheva et al. (2018) compared the access of patients with rare diseases to biotechnological drugs between several CEE countries in 2018, reporting that all these countries implemented special legislation for orphan drugs. The share of accessible orphan drugs as well as total expenditures varied across countries, being the highest in Greece and the lowest in Romania. The survey revealed some differences in the legal requirements for the pricing and reimbursement of biotechnological orphan medicinal products among the countries included in the study. All EU countries have developed and implemented pharmacoeconomic guidelines with or without some specific reimbursement requirements for orphan medicinal products. Cost-effectiveness analysis, cost-utility analysis, Markov models, meta-analysis, and discount rates for costs and outcomes were required only in Bulgaria, Poland, and Hungary. The access to orphan medicinal products was similar among the analyzed CEE countries, while the countries with the best access were Hungary and Greece.

Gammie et al. (2015) analyzed regulations and policies used by countries to allow patient access to orphan drugs in 2015 by performing a systematic review of evidence published between 1998 and 2014. They summarized the legislation of 35 countries from around the world, including 21 EU countries, and revealed that different types of special regulations for orphan drugs (national orphan drug policies, orphan drug designation, marketing authorization, marketing exclusivity, and tax credits) were present in most countries. A variation in the share of orphan drugs accessible for the patient was also observed.

A comparative analysis on the access to orphan drugs between the Balkan countries—five EU member states (Bulgaria, Croatia, Greece, Romania, and Slovenia) and two EU candidate countries (Serbia and Montenegro)—was performed by Pejcic et al. (2018). The review revealed significant inequalities among these states as well as an inadequate access to orphan drugs approved for the EU market; some improvement on the access for reimbursement and a better availability of orphan drugs for patients are needed.

Our study revealed that the great majority of the reimbursement recommendations were positive or conditional and about 65% of the considered orphan drugs were reimbursed from public funds. The agreement between the type of reimbursement recommendations and reimbursement status could be influenced by the bias between countries (differences in the decision-making process as well as legislation) and by the distributions of the reimbursement status. Hence, the presented coefficients should be treated as a descriptive statistic rather than an inference. In addition, the analysis could not be performed when no variation in the analyzed variable existed, like in the case of recommendations for metabolic drugs in Germany, while for 33 metabolic drugs the recommendations were presented for 13 drugs and all of them were positive. For this reason the agreement could not be calculated, as there were no reimbursed drugs with negative recommendations or negative recommendations and lack of reimbursement.

Our study has some limitations. First of all, we were not able to collect relevant data from some European countries, as they were not published online and they were also unavailable for the collaborating experts and as a result those countries were excluded from the analysis. Moreover, our data depicts the orphan designations identified until July 2018. Due to variations in both orphan drugs designations and reimbursement systems in the analyzed countries, the results of our study provide a snapshot of the situation and would need to be updated in future works. An ongoing monitoring of the reimbursement status of orphan drugs in analyzed countries and current trends in reimbursement decision-making for orphan drugs would be especially important, but requires a structural foundation beyond the abilities of this work. Also, although reimbursement status and recommendations were examined for 163 identified orphan drugs, it should be noted that not all of these orphan drugs are actually on the market in the selected countries. Cohen’s kappa coefficient of agreement could be calculated only for those countries that issued both positive and negative recommendations. In addition, the value of this coefficient could be affected by the prevalence of levels of the analyzed variables. Only in the cases where both recommendation and reimbursement status were available, coefficients were analyzed, which results in different sets of drugs used to calculate kappa coefficients in different countries. The kappa coefficient was close to 0 for Germany; this is due to the fact that there were only five negative results of the evaluation of additional benefit and all five drugs were finally reimbursed, giving 100% disagreement.

Despite those limitations, our study contributes significantly to the field of reimbursement of orphan drugs in European countries. Providing comprehensive and up-to-date information on access to reimbursed orphan drug and reimbursement policies, which may facilitate orphan drug management in these countries.

## Conclusions

About 65% of identified orphan drugs were reimbursed from public funds, and the majority of the reimbursement recommendations were found to be positive. Differences between countries were observed regarding the number of reimbursement recommendations for orphan drugs, the number of particular types of recommendations, and the number of reimbursed orphan drugs. The agreement between reimbursement recommendations and reimbursement status also differed between countries, but no patterns could be discerned. This study confirms the existence of equity gaps in orphan drug coverage, reflecting variability in both willingness and ability to pay.

## Data Availability Statement

The raw data supporting the conclusions of this manuscript will be made available by the authors, without undue reservation, to any qualified researcher.

## Author Contributions

ES, PK, RB, and AP contributed to the conception and/or design of the study. ES, PK, DP, HE, SS, AA, and MD contributed to data acquisition. ES, KPM, and PK contributed to data analysis and/or interpretation. ES, KPM, PK, and JS drafted the manuscript. All authors revised the manuscript critically for important intellectual content.

## Funding

This study was conducted within the statutory project: “Reimbursement and clinical aspects of orphan drug policy in Poland and Europe” (project number: K/ZDS/007863). We would like to thank Francis Arickx for help in data acquisition for Belgium.

## Conflict of Interest

SS is grant holder of a project on “How to reimburse and pay orphan drugs for rare diseases?” funded by the Scientific Research Foundation Flanders. 

The remaining authors declare that the research was conducted in the absence of any commercial or financial relationships that could be construed as a potential conflict of interest.
