# Supplementary figures and images for: Reimbursement Status and Recommendations Related to Orphan Drugs in European Countries
Source: Front Pharmacol. 2019 Nov 27;10:1279. doi: 10.3389/fphar.2019.01279 (PMC6890830; doi:10.3389/fphar.2019.01279)

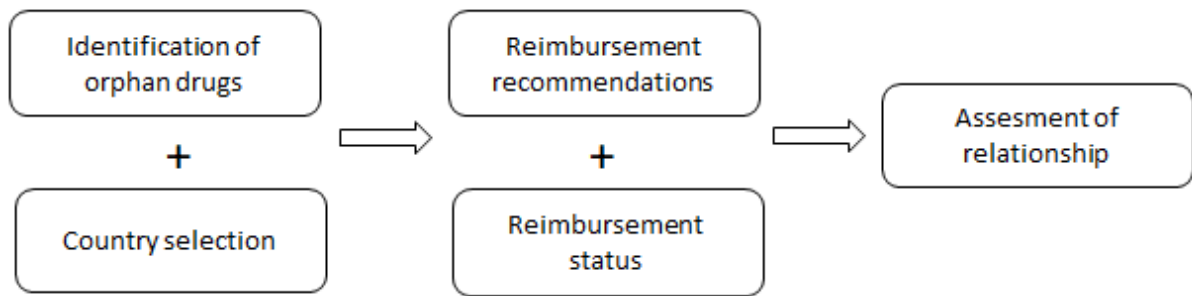

Supplement: Supplementary file 1 [file Image_1.pdf]
