# Supplementary material for: Reimbursement Status and Recommendations Related to Orphan Drugs in European Countries
Source: Front Pharmacol. 2019 Nov 27;10:1279. doi: 10.3389/fphar.2019.01279 (PMC6890830; doi:10.3389/fphar.2019.01279)
Supplement: Supplementary file 2 [file Table_1.pdf]

**Country**	**Reimbursement recommendations – source**	**Reimbursement status – sources**
England	National Institute for Health and Care Excellence (NICE)	National Health Service Drug Tariff
France	Transparency Committee at the French National Authority for Health (Haute Autorité de Santé, HAS)	Public drug database (Base de données publique des medicaments)
Germany	Federal Joint Committee (Gemeinsamer Bundesausschuss, G-BA)	-
Poland	Agency for Health Technology Assessment and Tariff System in Poland (Agencja Oceny Technologii Medycznych i Taryfikacji, AOTMiT)	Announcement of Minister of Health (Ministry of Health website)
Scotland	Scottish Medicines Consortium (SMC)	Scottish Drug Tariff
Spain	Health Technology Assessment Agency (Agencia de Evaluación de Tecnologias Sanitarias, AETS)	Medicine Online Information Center of AEMPS
Belgium	National Institute for Sickness and Disability Insurance (Institut national d'assurance maladie-invalidité, INAMI)	-
