# Supplementary material for: Reimbursement Status and Recommendations Related to Orphan Drugs in European Countries
Source: Front Pharmacol. 2019 Nov 27;10:1279. doi: 10.3389/fphar.2019.01279 (PMC6890830; doi:10.3389/fphar.2019.01279)
Supplement: Supplementary file 3 [file Table_2.pdf]

Tradename	Active substance	Indication
ADCETRIS	brentuximab vedotin	Treatment of adult patients with relapsed or refractory CD30+ Hodgkin lymphoma (HL): -following autologous stem cell transplant (ASCT) or -following at least two prior therapies when ASCT or multi-agent chemotherapy is not a treatment option. Treatment of adult patients with CD30+ HL at increased risk of relapse or progression following ASCT Treatment of adult patients with relapsed or refractory systemic anaplastic large cell lymphoma (sALCL). Treatment of adult patients with CD30+ cutaneous T-cell lymphoma (CTCL) after at least 1 prior systemic therapy.
ADEMPAS	riociguat	Treatment of adult patients with WHO Functional Class (FC) II to III with inoperable Chronic thromboembolic pulmonary hypertension (CTEPH), persistent or recurrent CTEPH after surgical treatment, to improve exercise capacity. As monotherapy or in combination with endothelin receptor antagonists, for the treatment of adult patients with pulmonary arterial hypertension (PAH) with WHO Functional Class (FC) II to III to improve exercise capacity. Efficacy has been shown in a PAH population including etiologies of idiopathic or heritable PAH or PAH associated with connective tissue disease.
ALOFISEL	darvadstrocel	Treatment of complex perianal fistulas in adult patients with non-active/mildly active luminal Crohn's disease, when fistulas have shown an inadequate response to at least one conventional or biologic therapy. Alofisel should be used after conditioning of fistula.
ALPROLIX	eftrenonacog alfa	Treatment and prophylaxis of bleeding in patients with haemophilia B (congenital factor IX deficiency). ALPROLIX can be used for all age groups.
ARZERRA	ofatumumab	In combination with chlorambucil or bendamustine, for the treatment of patients with chronic lymphocytic leukaemia (CLL) who have not received prior therapy and who are not eligible for fludarabine-based therapy. In combination with fludarabine and cyclophosphamide for the treatment of adult patients with relapsed CLL. Treatment of CLL in patients who are refractory to fludarabine and alemtuzumab.
BAVENCIO	avelumab	As monotherapy for the treatment of adult patients with metastatic Merkel cell carcinoma (MCC).
BESPONSA	inotuzumab ozogamicin	As monotherapy for the treatment of adults with relapsed or refractory CD22-positive B cell precursor acute lymphoblastic leukaemia (ALL). Adult patients with Philadelphia chromosome positive (Ph+) relapsed or refractory B cell precursor ALL should have failed treatment with at least 1 tyrosine kinase inhibitor (TKI).
BLINCYTO	blinatumomab	Treatment of adults with Philadelphia chromosome negative relapsed or refractory B -precursor acute lymphoblastic leukaemia (ALL).
BOSULIF	bosutinib	Treatment of adult patients with: - newly diagnosed chronic phase (CP) Philadelphia chromosome-positive chronic myelogenous leukaemia (Ph+ CML). - CP, accelerated phase (AP), and blast phase (BP) Ph+ CML previously treated with one or more tyrosine kinase inhibitor(s) [TKI(s)] and for whom imatinib, nilotinib and dasatinib are not considered appropriate treatment options.
BRINEURA	cerliponase alfa	Treatment of neuronal ceroid lipofuscinosis type 2 (CLN2) disease, also known as tripeptidyl peptidase 1 (TPP1) deficiency.
BRONCHITOL	mannitol	Treatment of cystic fibrosis (CF) in adults aged 18 years and above as an add-on therapy to best standard of care.
CARBAGLU	carglumic acid	Treatment of hyperammonaemia due to - isovaleric acidaemia, - methymalonic acidaemia, - propionic acidaemia.
CAYSTON	aztreonam	Suppressive therapy of chronic pulmonary infections due to Pseudomonas aeruginosa in patients with cystic fibrosis (CF) aged 6 years and older.
CEPLENE	histamine dihydrochloride	Maintainance therapy for adult patients with acute myeloid leukaemia in first remission concomitantly treated with interleukin-2 (IL-2). The efficacy of Ceplene has not been fully demonstrated in patients older than age 60.
CERDELGA	eliglustat	Long-term treatment of adult patients with Gaucher disease type 1 (GD1), who are CYP2D6 poor metabolisers (PMs), intermediate metabolisers (IMs) or extensive metabolisers (EMs)
CHENODEOXYCHOLIC ACID LEADIANT	chenodeoxycholic acid	Treatment of inborn errors of primary bile acid synthesis due to sterol 27 hydroxylase deficiency (presenting as cerebrotendinous xanthomatosis (CTX)) in infants, children and adolescents aged 1 month to 18 years and adults.
COAGADEX	human coagulation factor X	Treatment and prophylaxis of bleeding episodes and for perioperative management in patients with hereditary factor X deficiency.
COMETRIQ	cabozantinib	Treatment of adult patients with progressive, unresectable locally advanced or metastatic medullary thyroid carcinoma. For patients in whom Rearranged during Transfection (RET) mutation status is not known or is negative, a possible lower

		benefit should be taken into account before individual treatment decision.
CRESEMBA	isavuconazole	In adults for the treatment of: - invasive aspergillosis - mucormycosis in patients for whom amphotericin B is inappropriate
CRYSVITA	burosumab	Treatment of X-linked hypophosphataemia with radiographic evidence of bone disease in children 1 year of age and older and adolescents with growing skeletons.
CYSTADROPS	mercaptamine hydrochloride	Treatment of corneal cystine crystal deposits in adults and children from 2 years of age with cystinosis.
DACOGEN	decitabine	Treatment of adult patients aged 65 years and above with newly diagnosed de novo or secondary acute myeloid leukaemia (AML), according to the World Health Organization (WHO) classification, who are not candidates for standard induction chemotherapy.
DARZALEX	daratumumab	As monotherapy for the treatment of adult patients with relapsed and refractory multiple myeloma, whose prior therapy included a proteasome inhibitor and an immunomodulatory agent and who have demonstrated disease progression on the last therapy. In combination with lenalidomide and dexamethasone, or bortezomib and dexamethasone, for the treatment of adult patients with multiple myeloma who have received at least one prior therapy.
DEFITELIO	defibrotide	Treatment of severe hepatic veno- occlusive disease (VOD) also known as sinusoidal obstructive syndrome (SOS) in haematopoietic stem-cell transplantation (HSCT) therapy. It is indicated in adults and in adolescents, children and infants over 1 month of age.
DELTYBA	delamanib	Used as part of an appropriate combination regimen for pulmonary multi- drug resistant tuberculosis (MDR-TB) in adult patients when an effective treatment regimen cannot otherwise be composed for reasons of resistance or tolerability. Consideration should be given to official guidance on the appropriate use of antibacterial agents.
DINUTUXIMAB BETA APEIRON	dinutuximab beta	Treatment of high-risk neuroblastoma in patients aged 12 months and above, who have previously received induction chemotherapy and achieved at least a partial response, followed by myeloablative therapy and stem cell transplantation, as well as patients with history of relapsed or refractory neuroblastoma, with or without residual disease. Prior to the treatment of relapsed neuroblastoma, any actively progressing disease should be stabilised by other suitable measures. In patients with a history of relapsed/refractory disease and in patients who have not achieved a complete response after first line therapy, Dinutuximab beta Apeiron should be combined with interleukin-2 (IL-2).
ESBRIET	pirfenidone	In adults for the treatment of mild to moderate Idiopathic Pulmonary Fibrosis (IPF).
FARYDAK	panobinostat lactate anhydrous	In combination with bortezomib and dexamethasone, for the treatment of adult patients with relapsed and/or refractory multiple myeloma who have received at least two prior regimens including bortezomib and an immunomodulatory agent.
FIRAZYR	icatibant acetate	Symptomatic treatment of acute attacks of hereditary angioedema (HAE) in adults, adolescents and children aged 2 years and older, with C1-esterase- inhibitor deficiency.
FIRDAPSE	amifampridine	Symptomatic treatment of Lambert-Eaton myasthenic syndrome (LEMS) in adults.
GALAFOLD	migalastat	Long-term treatment of adults and adolescents aged 16 years and older with a confirmed diagnosis of Fabry disease (α- galactosidase A deficiency) and who have an amenable mutation.
GAZYVARO	obinutuzumab	In combination with chlorambucil, treatment of adult patients with previously untreated chronic lymphocytic leukaemia (CLL) and with comorbidities making them unsuitable for full-dose fludarabine based therapy. In combination with chemotherapy, followed by Gazyvaro maintenance therapy in patients achieving a response is indicated for the treatment of patients with previously untreated advanced follicular lymphoma. In combination with bendamustine followed by Gazyvaro maintenance is indicated for the treatment of patients with follicular lymphoma (FL) who did not respond or who progressed during or up to 6 months after treatment with rituximab or a rituximab-containing regimen.
GLYBERA	alipogene tiparvovec	For adult patients diagnosed with familial lipoprotein lipase deficiency (LPLD) and suffering from severe or multiple pancreatitis attacks despite dietary fat restrictions. The diagnosis of LPLD has to be confirmed by genetic testing. The indication is restricted to patients with detectable levels of LPL protein.
GRANUPAS	para-aminosali-cylic acid	Indicated for use as part of an appropriate combination regimen for multi-drug resistant tuberculosis in adults and paediatric patients from 28 days of age and

		older when an effective treatment regimen cannot otherwise be composed for reasons of resistance or tolerability. Consideration should be given to official guidance on the appropriate use of antibacterial agents.
HETLIOZ	tasimelteon	Treatment of Non-24-Hour Sleep-Wake Disorder (Non-24) in totally blind adults.
HOLOCLAR	ex vivo expanded autologous human corneal epithelial cells containing stem cells	Treatment of adult patients with moderate to severe limbal stem cell deficiency (defined by the presence of superficial corneal neovascularisation in at least two corneal quadrants, with central corneal involvement, and severely impaired visual acuity), unilateral or bilateral, due to physical or chemical ocular burns. A minimum of 1 - 2 mm2 of undamaged limbus is required for biopsy.
ICLUSIG	ponatinib	Indicated in adult patients with chronic phase, accelerated phase, or blast phase chronic myeloid leukaemia (CML) who are resistant to dasatinib or nilotinib; who are intolerant to dasatinib or nilotinib and for whom subsequent treatment with imatinib is not clinically appropriate; or who have the T315I mutation. Indicated in adult patients with Philadelphia chromosome positive acute lymphoblastic leukaemia (Ph+ ALL) who are resistant to dasatinib; who are intolerant to dasatinib and for whom subsequent treatment with imatinib is not clinically appropriate; or who have the T315I mutation.
IDELVION	albutrepenonacog alfa	Treatment and prophylaxis of bleeding in patients with haemophilia B (congenital factor IX deficiency). IDELVION can be used for all age groups.
IMBRUVICA	ibrutinib	As a single agent for the treatment of adult patients with relapsed or refractory mantle cell lymphoma (MCL). As a single agent for the treatment of adult patients with previously untreated chronic lymphocytic leukaemia (CLL). As a single agent or in combination with bendamustine and rituximab (BR) for the treatment of adult patients with CLL who have received at least one prior therapy. As a single agent for the treatment of adult patients with Waldenström's macroglobulinaemia (WM) who have received at least one prior therapy, or in first line treatment for patients unsuitable for chemo-immunotherapy.
IMNOVID	pomalidomide	In combination with dexamethasone, in the treatment of adult patients with relapsed and refractory multiple myeloma who have received at least two prior treatment regimens, including both lenalidomide and bortezomib, and have demonstrated disease progression on the last therapy.
INOVELON	rufinamide	Adjunctive therapy in the treatment of seizures associated with Lennox- Gastaut syndrome in patients aged 4 years and older.
JORVEZA	budesonide	Treatment of eosinophilic esophagitis (EoE) in adults (older than 18 years of age).
KALYDECO	ivacaftor	Treatment of patients with cystic fibrosis (CF) aged 6 years and older and weighing 25kg or more who have one of the following gating (class III) mutations in the CFTR gene:G551D, G1244E, G1349D, G178R, G551S, S1251N, S1255P, S549N or S549R. Treatment of patients with cystic fibrosis (CF) aged 18 years and older who have an R117H mutation in the CFTR gene
KANUMA	sebelipase alfa	Long-term enzyme replacement therapy (ERT) in patients of all ages with lysosomal acid lipase (LAL) deficiency
KETOCONAZOLE HRA	ketoconazole	Treatment of endogenous Cushing's syndrome in adults and adolescents above the age of 12 years.
KOLBAM	cholic acid	Treatment of inborn errors in primary bile acid synthesis due to sterol 27-hydroxylase (presenting ascerebrotendinous xanthomatosis, CTX) deficiency, 2- (or α-) methylacyl-CoA racemase (AMACR) deficiency or cholesterol 7α-hydroxylase (CYP7A1) deficiency in infants, children and adolescents aged 1 month to 18 years and adults.
KUVAN	sapropterin dihydrochloride	Treatment of hyperphenylalaninaemia (HPA) in adult and paediatric patients of all ages with phenylketonuria (PKU) who have been shown to be responsive to such treatment. Treatment of hyperphenylalaninaemia (HPA) in adult and paediatric patients with tetrahydrobiopterin (BH4) deficiency who have been shown to be responsive to such treatment.
KYPROLIS	carfilzomib	In combination with either lenalidomide and dexamethasone or dexamethasone alone is indicated for the treatment of adult patients with multiple myeloma who have received at least one prior therapy.
LAMZEDE	velmanase alfa	Enzyme replacement therapy for the treatment of non-neurological manifestations in patients with mild to moderate alpha mannosidosis.
LARTRUVO	olaratumab	In combination with doxorubicin for the treatment of adult patients with advanced soft tissue sarcoma who are not amenable to curative treatment with surgery or radiotherapy and who have not been previously treated with doxorubicin.

LEDAGA	chlormethine	Topical treatment of mycosis fungoides- type cutaneous T-cell lymphoma (MF- type CTCL) in adult patients.
LENVIMA	lenvatinib	Treatment of adult patients with progressive, locally advanced or metastatic, differentiated (papillary/follicular/Hürthle cell) thyroid carcinoma (DTC) refractory to radioactive iodine (RAI).
LUTATHERA	lutetium (177Lu) oxodotreotide	Treatment of unresectable or metastatic, progressive, well differentiated (G1 and G2), somatostatin receptor positive gastroenteropancreatic neuroendocrine tumours (GEP NETs) in adults.
LYNPARZA	olaparib	Monotherapy for the maintenance treatment of adult patients with platinum-sensitive relapsed BRCA-mutated (germline and/or somatic) high grade serous epithelial ovarian, fallopian tube, or primary peritoneal cancer who are in response (complete response or partial response) to platinum-based chemotherapy.
MEPACT	mifamurtide	In children, adolescents and young adults for the treatment of high-grade resectable non-metastatic osteosarcoma after macroscopically complete surgical resection. It is used in combination with post-operative multi- agent chemotherapy. Safety and efficacy have been assessed in studies of patients 2 to 30 years of age at initial diagnosis.
MOZOBIL	plerixafor	In combination with granulocyte-colony stimulating factor G-CSF to enhance mobilisation of haematopoietic stem cells to the peripheral blood for collection and subsequent autologous transplantation in patients with lymphoma and multiple myeloma whose cells mobilise poorly.
NATPAR	parathyroid hormone	Indicated as adjunctive treatment of adult patients with chronic hypoparathyroidism who cannot be adequately controlled with standard therapy alone.
NEXAVAR	sorafenib tosylate	Treatment of patients with progressive, locally advanced or metastatic, differentiated (papillary/ follicular/Hürthle cell) thyroid carcinoma, refractory to radioactive iodine.
NEXOBRID	concentrate of proteolytic en-zymes enriched in bromelain	Removal of eschar in adults with deep partial- and full-thickness thermal burns.
NINLARO	ixazomib	In combination with lenalidomide and dexamethasone is indicated for the treatment of adult patients with multiple myeloma who have received at least one prior therapy.
NPLATE	romiplostim	Indicated for adult chronic immune (idiopathic) thrombocytopenic purpura (ITP) patients who are refractory to other treatments (e.g. corticosteroids, immunoglobulins)
OCALIVA	obeticholic acid	Treatment of primary biliary cholangitis (also known as primary biliary cirrhosis) in combination with ursodeoxycholic acid (UDCA) in adults with an inadequate response to UDCA or as monotherapy in adults unable to tolerate UDCA.
OFEV	nintedanib	Treatment in adults of Idiopathic Pulmonary Fibrosis (IPF).
ONIVYDE	irinotecan hydrochloride trihydrate	Treatment of metastatic adenocarcinoma of the pancreas, in combination with 5-fluorouracil (5-FU) and leucovorin (LV), in adult patients who have progressed following gemcitabine based therapy.
OPSUMIT	macitentan	Used as monotherapy or in combination, for the long-term treatment of pulmonary arterial hypertension (PAH) in adult patients of WHO Functional Class (FC) II to III. Efficacy has been shown in a PAH population including idiopathic and heritable PAH, PAH associated with connective tissue disorders, and PAH associated with corrected simple congenital heart disease.
ORPHACOL	cholic acid	Treatment of inborn errors in primary bile acid synthesis due to 3beta- hydroxy-delta5-C27- steroid oxidoreductase deficiency or delta4-3- oxosteroid-5beta-reductase deficiency in infants, children and adolescents aged 1 month to 18 years and adults.
OXERVATE	cenegermin	Treatment of moderate (persistent epithelial defect) or severe (corneal ulcer) neurotrophic keratitis in adults.
PEYONA	caffeine citrate	Treatment of primary apnea of premature newborns.
PLENADREN	hydrocortisone	Treatment of adrenal insufficiency in adults.
PREVYMIS	letermovir	Prophylaxis of cytomegalovirus (CMV) reactivation and disease in adult CMV-seropositive recipients [R+] of an allogeneic haematopoietic stem cell transplant (HSCT). Consideration should be given to official guidance on the appropriate use of antiviral agents.

PROCYSBI	mercaptamine	Treatment of proven nephropathic cystinosis. Cysteamine reduces cystine accumulation in some cells (e.g. leukocytes, muscle and liver cells) of nephropathic cystinosis patients and, when treatment is started early, it delays the development of renal failure.
RAVICTI	glycerol phenylbutyrate	Indicated for use as adjunctive therapy for chronic management of adult and paediatric patients ≥2 months of age with urea cycle disorders (UCDs) including: deficiencies of carbamoyl phosphate- synthase-I (CPS) -ornithine carbamoyltransferase (OTC) - argininosuccinate synthetase (ASS), - argininosuccinate lyase (ASL) - arginase I (ARG) - ornithine translocase deficiency hyperornithinaemia -hyperammonaemia homocitrullinuria syndrome (HHH) Who cannot be managed by dietary protein restriction and/or amino acid supplementation alone. RAVICTI must be used with dietary protein restriction and, in some cases, dietary supplements (e.g., essential amino acids, arginine, citrulline, protein- free calorie supplements).
RAXONE	idebenone	Treatment of visual impairment in adolescent and adult patients with Leber's Hereditary Optic Neuropathy (LHON).
REVESTIVE	teduglutide	Treatment of patients aged 1 year and above with Short Bowel Syndrome. Patients should be stable following a period of intestinal adaptation after surgery.
REVLIMID	lenalidomide	Treatment of patients with transfusion- dependent anaemia due to low-or intermediate-1-risk myelodysplastic syndromes associated with an isolated deletion 5q cytogenetic abnormality when other therapeutic options are insufficient or inadequate. Treatment of adult patients with relapsed or refractory mantle cell lymphoma.
RYDAPT	midostaurin	In combination with standard daunorubicin and cytarabine induction and high dose cytarabine consolidation chemotherapy, and for patients in complete response followed by Rydapt single agent maintenance therapy, for adult patients with newly diagnosed acute myeloid leukaemia (AML) who are FLT3 mutation positive. As monotherapy for the treatment of adult patients with aggressive systemic mastocytosis (ASM), systemic mastocytosis with associated haematological neoplasm (SM AHN), or mast cell leukaemia (MCL).
SCENESSE	afamelanotide	Prevention of phototoxicity in adult patients with erythropoietic protoporphyria (EPP)
SIGNIFOR	pasireotide	Treatment of adult patients with Cushing's disease for whom surgery is not an option or for whom surgery has failed. Treatment of adult patients with acromegaly for whom surgery is not an option or has not been curative and who are inadequately controlled on treatment with another somatostatin analogue.
SIRTURO	bedaquiline	Used as part of an appropriate combination regimen for pulmonary multidrug-resistant tuberculosis (MDR-TB) in adult patients when an effective treatment regimen cannot otherwise be composed for reasons of resistance or tolerability. Consideration should be given to official guidance on the appropriate use of antibacterial agents.
SOLIRIS	eculizumab	Treatment of adults and children with : - Paroxysmal nocturnal haemoglobinuria (PNH). Evidence of clinical benefit is demonstrated in patients with haemolysis with clinical symptom(s) indicative of high disease activity, regardless of transfusion history. - atypical haemolytic uraemic syndrome (aHUS). -Refractory generalized myasthenia gravis (gMG) in patients who are anti-acetylcholine receptor (AChR) antibody-positive
SOMAKIT TOC	edotreotide	After radiolabelling with gallium (68Ga) chloride solution, the solution of gallium (68Ga) edotreotide obtained is indicated for Positron Emission Tomography (PET) imaging of somatostatin receptor overexpression in adult patients with confirmed or suspected well-differentiated gastro-enteropancreatic neuroendocrine tumours (GEP-NET) for localizing primary tumours and their metastases.
SPINRAZA	nusinersen sodium	Treatment of 5q Spinal Muscular Atrophy.
STRENSIQ	asfotase alfa	Long-term enzyme replacement therapy in patients with paediatric-onset hypophosphatasia to treat the bone manifestations of the disease.
STRIMVELIS	autologous CD34+ enriched cell fraction that contains CD34+ cells transduced with retroviral vector that	Treatment of patients with severe combined immunodeficiency due to adenosine deaminase deficiency (ADA-SCID), for whom no suitable human leukocyte antigen (HLA)-matched related stem cell donor is available.

	encodes for the human adenosine deaminase (ADA) cDNA sequence from human haematopoietic stem/progenitor (CD34+) cells	
SYLVANT	siltuximab	Treatment of adult patients with multicentric Castleman's disease (MCD) who are human immunodeficiency virus (HIV) negative and human herpesvirus-8 (HHV-8) negative.
TASIGNA	nilotinib	Treatment of adult and paediatric patients with newly diagnosed Philadelphia chromosome positive chronic myelogenous leukaemia (CML) in the chronic phase. Adult patients with chronic phase and accelerated phase Philadelphia chromosome positive CML with resistance or intolerance to prior therapy including imatinib. Efficacy data in patients with CML in blast crisis are not available. Paediatric patients with chronic phase Philadelphia chromosome positive CML with resistance or intolerance to prior therapy including imatinib.
TEPADINA	thiotepa	In combination with other chemotherapy medicinal products: 1) with or without total body irradiation (TBI), as conditioning treatment prior to allogeneic or autologous haematopoietic progenitor cell transplantation (HPCT) in haematological diseases in adult and paediatric patients; 2) when high dose chemotherapy with HPCT support is appropriate for the treatment of solid tumours in adult and paediatric patients.
THALIDOMIDE CELGENE	thalidomide	In combination with melphalan and prednisone as first line treatment of patients with untreated multiple myeloma, aged ≥ 65 years or ineligible for high dose chemotherapy. Thalidomide Celgene is prescribed and dispensed according to the Thalidomide Celgene Pregnancy Prevention Programme
TOBI PODHALER	tobramycin	Suppressive therapy of chronic pulmonary infection due to Pseudomonas aeruginosa in adults and children aged 6 years and older with cystic fibrosis. Consideration should be given to official guidance on the appropriate use of antibacterial agents.
TORISEL	temsirolimus	Treatment of adult patients with relapsed and / or refractory mantle cell lymphoma (MCL).
TRANSLARNA	ataluren	Treatment of Duchenne muscular dystrophy resulting from a nonsense mutation in the dystrophin gene, in ambulatory patients aged 5 years and older. Efficacy has not been demonstrated in non-ambulatory patients. The presence of a nonsense mutation in the dystrophin gene should be determined by genetic testing.
VENCLYXTO	venetoclax	As monotherapy for the treatment of chronic lymphocytic leukaemia (CLL) in the presence of 17p deletion or TP53 Mutation in adult patients who are unsuitable for or have failed a B-cell receptor pathway inhibitor. As monotherapy for the treatment of CLL in the absence of 17p deletion or TP53 mutation in adult patients who have failed both chemoimmunotherapy and a B -cell receptor pathway inhibitor.
VIDAZA	azacitidine	Treatment of adult patients who are not eligible for haematopoietic stem cell transplantation with: - intermediate-2 and high-risk myelodysplastic syndromes (MDS) according to the International Prognostic Scoring System (IPSS), - chronic myelomonocytic leukaemia (CMML) with 10-29% marrow blasts without myeloproliferative disorder, - acute myeloid leukaemia (AML) with 20- 30 % blasts and multi-lineage dysplasia, according to World Health Organisation (WHO) classification. Treatment of adult patients aged 65 years or older who are not eligible for HSCT with AML with >30% marrow blasts according to the WHO classification.
VIMIZIM	elosulfase alfa	Treatment of mucopolysaccharidosis, type IVA (Morquio A Syndrome, MPS IVA) in patients of all ages.
VOLIBRIS	ambrisentan	Treatment of pulmonary arterial hypertension (PAH) in adult patients of WHO Functional Class (FC) II to III, including use in combination treatment. Efficacy has been shown in idiopathic PAH (IPAH) and in PAH associated with connective tissue disease.
VOTUBIA	everolimus	Treatment of adult patients with renal angiomyolipoma associated with tuberous sclerosis complex (TSC) who are at risk of complications (based on factors such as tumour size or presence of aneurysm, or presence of multiple or bilateral tumours) but who do not require immediate surgery.The evidence is based on analysis of

		change in sum of angiomyolipoma volume. Treatment of patients with subependymal giant cell astrocytoma (SEGA) associated with tuberous sclerosis complex (TSC) who require therapeutic intervention but are not amenable to surgery. The evidence is based on analysis of change in SEGA volume. Further clinical benefit, such as improvement in disease-related symptoms, has not been demonstrated.
VPRIV	velaglucerase alfa	Long-term enzyme replacement therapy (ERT) in patients with type 1 Gaucher disease.
VYNDAQEL	tafamidis	Treatment of transthyretin amyloidosis in adult patients with stage 1 symptomatic polyneuropathy to delay peripheral neurologic impairment.
WAKIX	pitolisant	Treatment in adults of narcolepsy with or without cataplexy.
XALUPRINE	mercaptopurine	Treatment of acute lymphoblastic leukaemia (ALL) in adults, adolescents and children.
XERMELO	telotristat	Treatment of carcinoid syndrome diarrhoea in combination with somatostatin analogue (SSA) therapy in adults inadequately controlled by SSA therapy.
YONDELIS	trabectedin	In combination with pegylated liposomal doxorubicin (PLD), treatment of patients with relapsed platinum-sensitive ovarian cancer.
ZALMOXIS	allogeneic T cells genetically modified with a retroviral vector encoding for a truncated form of the human low affinity nerve growth factor receptor (ΔLNGFR) and the herpes simplex I virus thymidine kinase (HSV-TK Mut2)	Indicated as adjunctive treatment in haploidentical haematopoietic stem cell transplantation (HSCT) of adult patients with high-risk haematological malignancies.
ZAVESCA	miglustat	Treatment of progressive neurological manifestations in adult patients and paediatric patients with Niemann-Pick type C disease.
ZEJULA	niraparib	As monotherapy for the maintenance treatment of adult patients with platinum sensitive relapsed high grade serous epithelial ovarian, fallopian tube, or primary peritoneal cancer who are in response (complete or partial) to platinum based chemotherapy.
JAKAVI	ruxolitinib	Treatment of chronic idiopathic myelofibrosis. Treatment of myelofibrosis secondary to polycythaemia vera or essential thrombocythaemia
SUTENT	sunitinib	Treatment of malignant gastrointestinal stromal tumours. Treatment of renal cell carcinoma
EVOLTRA	clofarabine	Treatment of acute lymphoblastic leukaemia
UPTRAVI	selexipag	Treatment of pulmonary arterial hypertension and chronic thromboembolic pulmonary hypertension
LUMACAFTOR	lumacaftor	Treatment of cystic fibrosis
EXJADE	deferasirox	Treatment of chronic iron overload requiring chelation therapy
GLIOLAN	5-Aminolevulinic acid hydrochloride	Intra-operative photodynamic diagnosis of residual glioma
REPLAGAL	Alpha-galactosidase A	Treatment of Fabry disease
FABRAZYME	Alpha-galactosidase A	Treatment of Fabry disease
XAGRID	Anagrelide hydrochloride	Treatment of essential thrombocythaemia
TRISENOX	Arsenic trioxide	Treatment of acute promyelocytic leukaemia
LYMPREVA	Autologous tumor-derived immunoglobulin idiotype coupled to keyhole limpet	Treatment of follicular lymphoma

	haemocyanin	
INLYTA	Axitinib	Treatment of renal-cell carcinoma
CYSTADANE	Betaine anhydrous	Treatment of homocystinuria
TRACLEER	Bosentan	Treatment of pulmonary arterial hypertension and chronic thromboembolic pulmonary hypertension. Treatment of systemic sclerosis (scleroderma)
BUSILVEX	Busulfan	Conditioning treatment prior to haematopoietic-progenitor-cell transplantation
ONSENAL	Celecoxib	Treatment of familial adenomatous polyposis (FAP)
UNITUXIN	Chimeric monoclonal antibody against GD2(dinutuximab)	Treatment of neuroblastoma
VERKAZIA	Ciclosporin	Treatment of vernal keratoconjunctivitis
LITAK	Cladribine	Treatment of indolent non-Hodgkin's lymphoma
DROPCYS	Cysteamine hydrochloride	Treatment of cystinosis
SPRYCEL	Dasatinib	Treatment of acute lymphoblastic leukaemia. Treatment of chronic myeloid leukaemia
NEOFORDEX	Dexamethasone (40 mg tablet)	Treatment of multiple myeloma
SAVENE	Dexrazoxane	Treatment of anthracycline extravasations
REVOLADE	Eltrombopag olamine	Treatment of idiopathic thrombocytopenic purpura
AFINITOR	Everolimus	Treatment of renal cell carcinoma
NEOCEPRI	Folic acid to be used with N-[4-[[(2-amino-3,4-dihydro-4-oxo-6-pteridinyl)methyl]amino]benzoyl]-D-gamma-glutamyl-(2S)-2-amino-beta-alanyl-L-alpha-aspartyl-L-cysteine	Diagnosis of positive folate-receptor status in ovarian cancer
SIKLOS	Hydroxyurea	Treatment of sickle cell syndrome
PEDEA	Ibuprofen	Treatment of patent ductus arteriosus
ELAPRASE	Iduronate-2-sulfatase	Treatment of mucopolysaccharidosis, type II (Hunter Syndrome)
VENTAVIS	Iloprost	Treatment of primary and of the following forms of secondary pulmonary hypertension…
GLIVEC	Imatinib mesilate	Treatment of dermatofibrosarcoma protuberans Treatment of acute lymphoblastic leukaemia Treatment of chronic eosinophilic leukaemia and the hypereosinophilic syndrome Treatment of chronic myeloid leukaemia Treatment of malignant gastrointestinal stromal tumours Treatment of myelodysplastic / myeloproliferative diseases
ALDURAZYME	laronidase	Treatment of mucopolysaccharidosis type I
ORKAMBI	Lumacaftor / ivacaftor	Treatment of cystic fibrosis
INCRELEX	Mecasermin	Treatment of primary insulin-like growth factor-1 deficiency due to molecular or genetic defects
CORLUXIN	Mifepristone	Treatment of hypercortisolism (Cushing's syndrome) of endogenous origin
LYSODREN	Mitotane	Treatment of adrenal cortical carcinoma
FOLCEPRI	etarfolatide	Diagnosis of positive folate-receptor status in ovarian cancer
NAGLAZYME	galsulfase	Treatment of mucopolysaccharidosis VI (MPS VI) or Maroteaux-Lamy syndrome
ATRIANCE	Nelarabine	Treatment of acute lymphoblastic leukaemia
ORFADIN	Nitisinone	Treatment of tyrosinaemia type 1

SOMAVERT	Pegvisomant	Treatment of acromegaly
PHOTOBARR	Porfimer sodium (for use with photodynamic therapy)	Treatment of high-grade dysplasia in Barrett's oesophagus
CYRAMZA	Ramucirumab	treatment of gastric cancer
ELOCTA	efmoroctocog alfa	Treatment of haemophilia A
MYOZYME	alglucosidase alfa	Treatment of glycogen storage disease type II (Pompe's disease)
NOVOTHIRTEEN	catridecacog	Treatment of hereditary factor-XIII deficiency
ILARIS	canakinumab	Treatment of cryopirin-associated periodic syndromes (FCUS, MWS, and NOMID)
OBIZUR	susoctocog alfa	Treatment of haemophilia A
RILONACEPT REGENERON	Rilonacept	Treatment of cryopirin-associated periodic syndromes
RUBRACA	Rucaparib	Treatment of ovarian cancer
REVATIO	Sildenafil citrate	Treatment of pulmonary arterial hypertension and chronic thromboembolic pulmonary hypertension
THELIN	Sitaxentan sodium	Treatment of pulmonary arterial hypertension and chronic thromboembolic pulmonary hypertension
XYREM	Sodium oxybate	Treatment of narcolepsy
DIACOMIT	Stiripentol	Treatment of severe myoclonic epilepsy in infancy
DIACOMIT	vintafolide	Treatment of severe myoclonic epilepsy in infancy
PRIALT	Ziconotide (intraspinal use)	Treatment of chronic pain requiring intraspinal analgesia
WILZIN	Zinc acetate dihydrate	Treatment of Wilson’s disease
